# The Incidence, Mortality, and Survival Trends of Pancreatic Cancer in Kazakhstan: Data from the National Electronic Registry of Oncological Patients (2014–2023)

**DOI:** 10.3390/cancers17142277

**Published:** 2025-07-08

**Authors:** Aigerim Biniyazova, Ruslan Akhmedullin, Ayana Ablayeva, Altynay Beyembetova, Diyora Abdukhakimova, Abzal Zhumabekov, Temirgali Aimyshev, Gulnur Zhakhina, Temirlan Seyil, Yuliya Semenova, Abduzhappar Gaipov

**Affiliations:** 1 Department of Medicine, School of Medicine, Nazarbayev University, Astana 010000, Kazakhstan; aigerim.biniyazova@nu.edu.kz (A.B.); ruslan.akhmedullin@nu.edu.kz (R.A.); ayana.ablayeva@nu.edu.kz (A.A.); altynay.akhmedinova@nu.edu.kz (A.B.); dabdukhakimova@nu.edu.kz (D.A.); temirgali.aimyshev@nu.edu.kz (T.A.); gulnur.zhakhina@nu.edu.kz (G.Z.); temirlan.seyil@nu.edu.kz (T.S.); 2 Department of Neurosurgery, National Hospital of the Medical Center of the Presidential Administration of the Republic of Kazakhstan, Almaty 050000, Kazakhstan; zhumabekov_a@snh.kz; 3 Department of Surgery, School of Medicine, Nazarbayev University, Astana 010000, Kazakhstan; yuliya.semenova@nu.edu.kz; 4 Clinical Academic Department of Internal Medicine, University Medical Center, Astana 010000, Kazakhstan

**Keywords:** PC, incidence, prevalence, mortality, Kazakhstan

## Abstract

**Background:** Pancreatic cancer (PC) has an extremely poor prognosis and is growing worldwide. In Kazakhstan, it is among the five leading causes of cancer death. However, local epidemiological studies of PC are scarce. A retrospective population-based study was conducted to investigate the PC incidence, mortality, and survival in Kazakhstan, using data from the Electronic Registry of Oncological Patients for the period 2014–2023. **Methods:** Incidence, prevalence, and crude mortality rates were calculated per 100,000 population. The all-cause mortality rate per 1000 person-years (PY) was obtained based on socio-demographic and medical characteristics and for different regions. Survival analysis was performed using the Kaplan–Meier method and Cox regression models. **Results:** A total of 11,934 cases were analyzed. The annual incidence rate significantly increased from 5.9 to 6.9 per 100,000. The mortality rate per 100,000 also increased from 4 to 6. The highest incidence was observed in the 60–74 age group (49.2%), with an equal sex distribution. The mortality rate was the highest in patients aged 75 and older. Northern and central regions had the highest incidence rates as of 2023. Atyrau, West Kazakhstan, Pavlodar, and Abay had the highest mortality rates per 1000 PY. The five-year survival rate was 10.9%. An older age, a male sex, advanced cancer stages, and a Russian ethnicity significantly increased the risk of death. **Conclusions:** PC represents a significant healthcare problem in Kazakhstan, with late diagnosis and poor survival being the main challenges. This study highlights the need to improve the timely detection of PC and address the identified disparities.

## Simple Summary

Pancreatic cancer (PC) continues to be a significant health issue worldwide, causing a heavy burden due to its high mortality. Our study revealed the global trends in Kazakhstan, as well as local regional and ethnic characteristics. The study revealed an increasing incidence of and mortality from PC in 2014–2023, very low survival rates, late diagnoses, and significant inequalities based on different demographic and clinical characteristics. This study highlights the urgent need for multilateral interventions in Kazakhstan in PC diagnostics and care.

## 1. Introduction

Pancreatic cancer (PC) is an aggressive type of cancer with very low survival rates. With a median age of detection of 71 years, it primarily affects older adults, impacting men and women equally [1,2,3]. The GLOBOCAN 2022 stated that PC is the 12th most common cancer and the 6th most deadly cancer worldwide [4]. In 2022, the number of new cases reached 510,992 worldwide, of which 467,409 died. The disease is mainly prevalent in Asia, Europe, and North America, with the highest burden observed in Asian countries, accounting for nearly 50% of all cases globally [4].

Kazakhstan is a Central Asian country with the fastest-growing economy in the region. After the collapse of the USSR, the country’s healthcare system operated on the Beveridge model. In the beginning of 2020, it switched to a social insurance system [5]. Oncological care in the country is included in the guaranteed volume of free medical care (GVFMC), which encompasses diagnostics, treatment, and palliative care. The compulsory social health insurance (CSI) system also covers a significant part of oncological care and expands the access to treatment. In the case of preventive screening examinations and suspicions of malignant neoplasms, diagnostic tests are carried out within the framework of the GVFMC and the CSI system [6].

According to the World Health Organization [7], cancer is one of the main health problems in Kazakhstan. In 2021, 14,004 people died from cancer, which ranks fourth in mortality among non-communicable diseases. Among all the types of cancer, PC ranks fifth in mortality, with about 900 deaths a year. More than 1100 new cases are diagnosed annually, and there is a tendency for its incidence to increase [8,9,10]. The greatest number of deaths occurs among elderly individuals, affecting both men and women equally. The situation regarding the timing of diagnosis is similar to the global trends: in a number of regions, the frequency of the diagnosis of PC at early stages is quite low (26%). Metastatic forms are detected in one-third of cases [10].

While the annual increase in PC-related rates is higher in Kazakhstan than any other Asian country [8], obtaining further evidence from nationwide studies remains essential. Comprehensive nationwide epidemiological studies are critical to understanding the burden and long-term trends of PC in the country. The current literature is limited to a handful of studies where the authors studied the epidemiology of PC in the countries of the former Soviet Union or the Asia–Pacific region using secondary data from the Global Burden of Disease (GBD) study or GLOBOCAN [11,12,13]. The most recent epidemiological study by local authors, covering the period of 2018–2020, was published in 2022 based on data from the National Oncology Center of Kazakhstan [10]. Another recent study showed the mortality trends for common cancer types in Kazakhstan for 2014–2022 [14].

This study aimed to analyze nationwide data on PC from the Electronic Registry of Oncological Patients (EROP), a part of the Unified National Electronic Health System (UNEHS) of Kazakhstan, for the period between 2014 and 2023. The incidence, prevalence, and mortality trends of PC were assessed for the decade, and survival analysis was conducted to explore the impact of patients’ socio-demographic and medical characteristics on their survival. The incidence rates and all-cause mortality rates per 1000 PY were obtained for each of the regions of Kazakhstan, which are the administrative and territorial units of the country.

## 2. Methods

### 2.1. Study Design and Population

**Figure 1:**
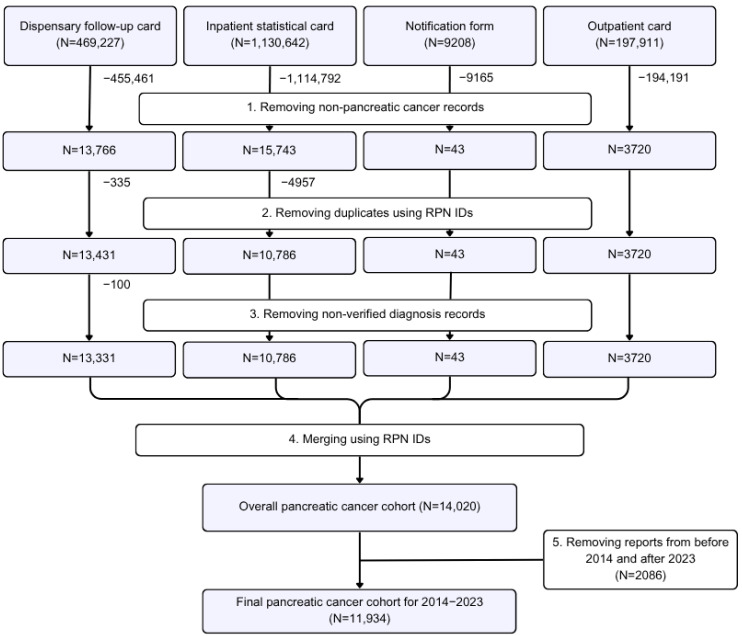
Scheme showing the preprocessing of the data in the EROP and filtering of the PC sample. The arrows indicate the flow of data and the application of each step. The white boxes with numbers represent the main stages of processing.

In this retrospective cohort study, anonymized data on PC patients registered between 2014 and 2023 were obtained from the Electronic Registry of Oncological Patients (EROP) of Kazakhstan, in which each patient is assigned a unique registration serial number (RPN ID). The registry comprises four different outpatient and inpatient documentation forms (dispensary follow-up cards, outpatient cards, statistical cards of discharged patients, and notification forms) [15]. The database preprocessing and cohort filtering processes are presented in Figure 1. After obtaining the initial PC sample using the forms, duplicates were removed using the RPN IDs. The first cases registered were left in the cohort. Then unverified observations were deleted based on the deregistration reason. The merging of the forms into one single dataset was carried out using the RPN IDs. The earliest dates on the forms were considered the first event dates. Some of the missing data on patients’ age (3.7%), sex (0.5%), ethnicity (0.2%), place of residence (3%), and region (94.5%) were obtained from the Electronic Registry of Dispensary Patients (ERDP), another part of the UNEHS [16]. For this purpose, the PC cohort in the ERDP was filtered and preprocessed in the same way as that in the EROP and the variables were merged with those in the EROP dataset using the RPN IDs.

### 2.2. Study Sample

The EROB was commissioned in 2013 and had reached its full capacity by 2014. For this research, it was accessed in November 2024. Therefore, our study included the latest available data on PC cases diagnosed between 2014 and 2023 (inclusive). For cohort selection, the 10th International Classification of Diseases (ICD-10) was used (PC codes from “C25” to “C25.9” and the code for benign neoplasms of the pancreas, “D37.7”). In the selected cohort, we studied the following variables: the patients’ age at diagnosis, sex, ethnicity, region within the country, place of residence, stage at diagnosis, ICD-10 code for a tumor location within the pancreas, and survival outcome. The patients were divided into the following age categories: 0–29, 30–44, 45–59, 60–74, and ≥75 years old. For the age, sex, stage at diagnosis, ethnicity, and region variables, the missing value proportions were 1.8%, 5.3%, 3.1%, 5.3%, and 1%, respectively. STATA included only the complete cases in the statistical analyses, automatically excluding rows with missing data. Imputation was not used due to the low proportion of missingness. The place of residence data was incomplete for 52.3% of the patients; therefore, it was excluded from all the analyses. Descriptive statistics were calculated for the complete cases.

### 2.3. Outcomes

Crude incidence, prevalence, and all-cause mortality rates were calculated per 100,000 annual average population. Age-specific incidence and mortality rates were calculated based on the population size of the corresponding age group. Demographic data were taken from demographic yearbooks published by the National Statistical Bureau of Kazakhstan [17]. In the survival analysis, the patients were tracked from their initial diagnosis date to their death or the study’s conclusion date, 31 December 2023.

### 2.4. Statistical Analysis

The statistical software STATA v. 16 MP2 (STATA Corporation, College Station, TX, USA) was used for data cleaning, management, and statistical analysis. The descriptive statistics of the sample (medical and demographic characteristics) were calculated as means and percentages. To assess the differences between the patient groups, a two-sample *t*-test was used for the continuous variables, with the survival outcome as the dependent variable. To examine the association between the categorical variables and survival outcomes, a chi-square test was performed. The survival probability was estimated using Kaplan–Meier analysis, and a log-rank test was used for statistical comparisons between the groups. Cox proportional hazard regression analysis was used to obtain the crude and adjusted hazard ratios. Two models were constructed using Cox regression: model 1 was constructed to obtain crude hazard ratios for the age, sex, ethnicity, and cancer stage variables. In model 2, all the variables were modeled at once (adjusted) to account for their impacts on each other. The models’ fit was assessed using the Schoenfeld test. The variance inflation factor (VIF) was employed to detect multicollinearity, which was evident for the age and cancer stage variables. Further, to identify the isolated effect of these variables, we centered the age variable and rescaled it by 10 years in order to obtain meaningful estimates. The level of statistical significance was set at a *p*-value of 0.05. The all-cause mortality rate per 1000 person-years (PY) was determined based on patients’ medical and demographic characteristics and regions. The choropleth maps were created using Datawrapper, a web-based data visualization tool (Datawrapper GmbH, Berlin, Germany). This study was conducted according to the STROBE guidelines for cohort studies [18].

## 3. Results

### 3.1. Baseline Characteristics

A total of 11,934 PC cases were identified and analyzed according to the inclusion criteria. The medical and demographic characteristics of the patients with true values are shown in Table 1. The mean age of the patients at diagnosis was 64.5 ± 11.3 years, ranging from 4 to 102 years. In general, the disease affected men and women equally (49.9% and 50.1%, respectively). The highest numbers of cases were recorded in the 60–74 age group (50.1%), and among Kazakhs (52.7%). The overall mortality in the cohort was 84.9%. The median follow-up time was 4.6 years from the time of diagnosis (IQR: 2.25–7.14). In 74.3% of the patients, the disease was inoperable (stages III and IV). The most common tumor site was the pancreatic head (59.6%), followed by the body (13.4%) and tail of the pancreas (9%).

### 3.2. Incidence, Prevalence and Mortality Trends over Time

**Figure 2:**
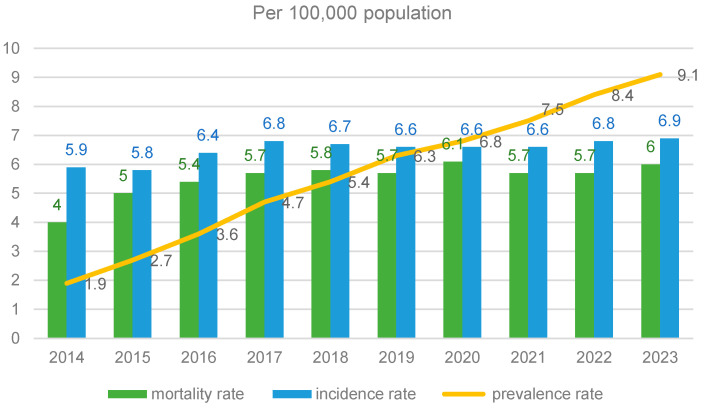
PC rates over the period studied.

The incidence, prevalence, and all-cause mortality rates for the period studied were estimated, and the figures are shown in Figure 2. The incidence of PC per 100,000 population rose from 5.9 in 2014 to 6.9 in 2023. The prevalence rate rose from 1.9 per 100,000 population in 2014 to 9.1 in 2023. The all-cause mortality rate increased from 4 per 100,000 in 2014 to 6 in 2023. After increasing to 6.8 per 100,000 in 2017, the incidence rate remained relatively stable for the rest of the period. A mild decrease in the PC mortality rates could be seen in 2021 and 2022.

**Figure 3:**
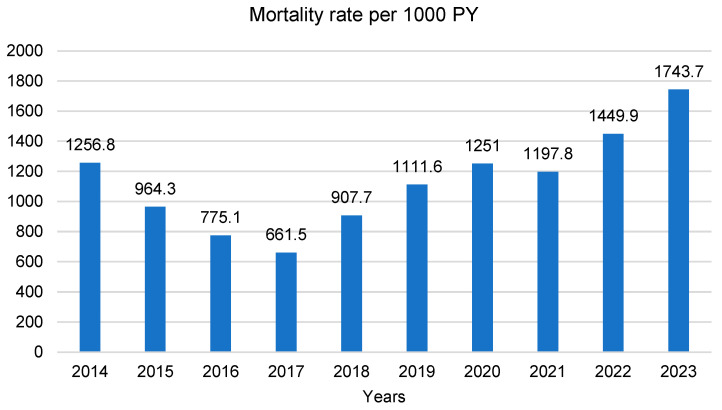
All-cause mortality rate of PC patients per 1000 PY 2014–2023.

The all-cause mortality rate of PC patients per 1000 PY is shown in Figure 3. It decreased from 1256.8 in 2014 to 661.5 in 2017; then it showed an increasing trend, except for a mild decrease in 2021, and finally reached 1743.7 in 2023. An older age, male sex, Russian ethnicity, advanced stage, and tumor location in the endocrine part of the pancreas increased the mortality of the patients studied (Table 1).

**Figure 4:**
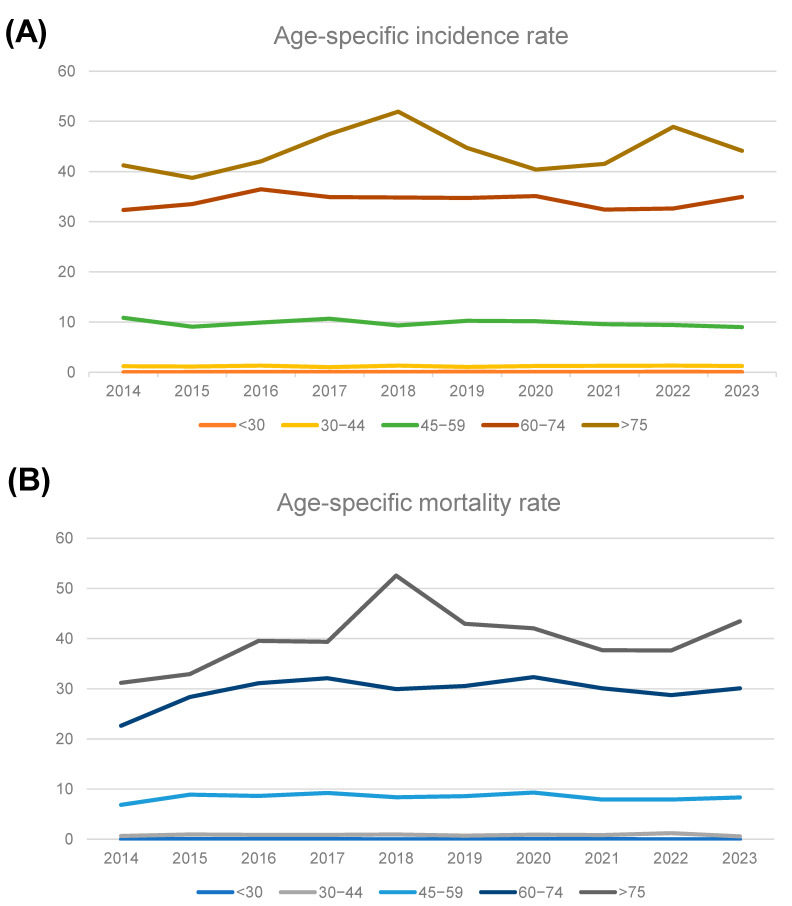
Age-specific incidence (**A**) and mortality rates (**B**) of PC patients per 100,000 population between 2014 and 2023.

The age-specific incidence and mortality rates for PC in Kazakhstan are presented in Figure 4. The age-specific incidence of PC per 100,000 increased significantly during this period in the 60–74 age category (from 32.3 in 2014 to 35 in 2023) and in those over 75 years old (from 41.2 in 2014 to 44.2 in 2023). The mortality rates also showed an upward trend in all the over-45 age categories. The largest increase in the mortality was observed among patients aged 75 years and older, where the rates increased sharply from 31.2 in 2014 to 43.4 in 2023.

**Figure 5:**
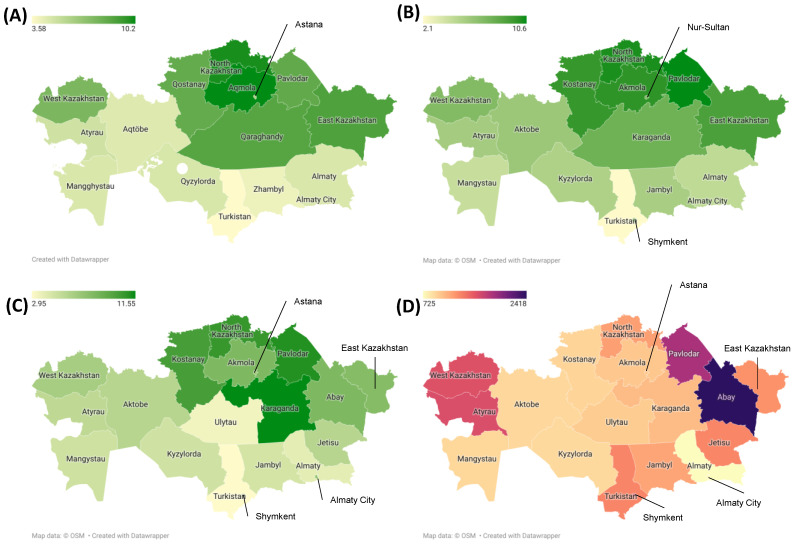
PC incidence rate per 100,000 population in 2014–2017 (**A**), 2018–2021 (**B**), 2022–2023 (**C**), and mortality rate per 1000 PY (**D**) in each region of Kazakhstan.

Figure 5 illustrates the incidence rate of PC in the different regions of Kazakhstan in 2014–2017, 2018–2021, and 2022–2023 and the mortality rate per 1000 PY. The incidence rate in each of the regions by year is shown in Supplementary Figure S1. According to the data, the northern and eastern regions had higher incidence rates during this period compared to the southern and western regions. Most regions showed an increasing trend in their incidence rates, except for Turkistan. By the end of this period, the Karaganda, Kostanay, North Kazakhstan, and Pavlodar regions had higher incidence rates for PC compared to the other regions. The distribution of the mortality rate per 1000 PY shows that the Atyrau, West Kazakhstan, Pavlodar, and Abay regions were characterized by the highest mortality per 1000 PY.

### 3.3. Survival Analysis

The Kaplan–Meier estimates showed a 5-year survival rate of 10.9% (95% CI, 10.31–11.56) and a median survival of 2.86 months (IQR: 2.73–2.99). A graphical illustration of the survival by patients’ age group, sex, ethnicity, and stage at diagnosis is presented in Figure S2. The differences between the groups were significant (*p* < 0.05 for log-rank test). According to them, the survival probability was lower in older age groups, male patients, patients of Russian ethnicity, and those diagnosed at late stages. The results of the Cox proportional hazards regression analyses are presented in Table 2. Overall, significant effects of patients’ age, sex, ethnicity, and stage at diagnosis on the risk of death were found. Age was found to be a significant predictor of death: in the adjusted model, each 10-year increment in age increased the hazard of death by 21% (aHR = 1.21, 95% CI of 1.19–1.24). The risk of death was 17% higher for male patients than for female patients (aHR = 1.17, 95% CI of 1.06–1.20). A Russian ethnicity was associated with a 13% increase in the risk of death relative to that of the “other” ethnic group (aHR = 1.13, 95% CI of 1.06–1.20). The risk of death increased one and a half times with each increase in the stage, reaching 4.80 at stage IV (aHR = 4.80, 95% CI of 4.13–5.59).

## 4. Discussion

This 10-year epidemiological study was the first in-depth and thorough work on PC in Kazakhstan that utilized empirical data from the Electronic Registry of Oncological Patients. The incidence, prevalence, and mortality trends of PC were examined for the past decade, and, for the first time, the survival of patients with PC in Kazakhstan was analyzed. Overall, this study revealed a significant increase in both the PC incidence and mortality during the study period, along with late diagnoses and low survival. The patient’s age, a male sex, a Russian ethnicity, and the stage at diagnosis were found to be significant predictors of mortality.

The results of our study are in line with the global trends and show an increase in the PC incidence and mortality over the past decade [4]. The incidence of PC in Kazakhstan increased from 5.9 per 100,000 population in 2014 to 6.9 in 2023, which aligns with the world literature [19]. In 2020, the global incidence rate was 6.4 per 100,000, while in Europe it was 18.7 per 100,000 [20]. Thus, the incidence rate of PC in Kazakhstan is comparable to the world average. In 2020, the mortality rate for PC was 6 deaths per 100,000 worldwide and 17.6 per 100,000 in Europe [20]. In Kazakhstan, this rate was 6 in 2023. Among European countries, the incidence and mortality rates in Kazakhstan are comparable to those of Ireland, which reports the lowest rates in Europe [20]. In Asia, the crude incidence and mortality rates are equal to 4.7 and 4.4 per 100,000, respectively [12].

The increasing trend in the PC incidence worldwide may be due to the population aging and lifestyle changes [12,21]. Supporting this, Siegel et al. reported an increase in the PC incidence among older adults [19]. Another study found that more than 80% of PC cases are diagnosed in individuals aged 60 to 80 [22]. In Asia and Oceania, most individuals with PC are over 65, constituting 62% and 70% of those with PC, respectively [23]. In the United States, the patterns are similar to this: PC mostly affects people aged 65–74, and the mortality is also highest among individuals in this age group [24]. In our study, more than 70% of cases were attributed to individuals aged 60 and older, with a mean age of 64.5 at diagnosis.

Globally, the PC incidence is usually slightly male-dominated, especially among those under 75 years of age. This sex disparity can be attributed to factors such as smoking and physiological differences [8]. But in recent years, there has been an increasing trend in the incidence among women. This can be attributed to the increasing prevalence of obesity, diabetes, and alcohol consumption among them [2]. The mortality rate has also gradually increased each year compared to that of other cancers [3]. In 1990, PC was the ninth leading cause of death among all cancers, but it increased to the rank of sixth in 2021 [25]. It is projected that PC will become the second leading cause of cancer death in developed countries by 2030 [1,2].

According to our results, the incidence rate was lower during 2020 and 2021 compared to 2022, while the crude mortality rate decreased slightly from 6.1 in 2020 to 5.7 in 2021 and 2022. At the beginning of the COVID-19 pandemic, many cancers showed a decrease in their incidence, mainly due to a reduction in screening; however, no association was observed between the COVID-19 pandemic and PC incidence [26]. In the Netherlands, the pandemic had little impact on the treatment and outcomes of PC, showing a comparable incidence in the second quarter of 2020 with that in previous years. However, in the fourth quarter of 2020, the incidence increased due to growth in the proportion of metastatic cases diagnosed [27]. England also reported no significant impact of the COVID-19 pandemic on the incidence rate [28], while in Japan the total number of cases slightly decreased by 1.9% in 2020 compared to the number in 2019. Similarly to in the Netherlands, more advanced-stage cases were observed than in 2019 [29]. Regarding mortality, it can be assumed that the increase in the number of advanced disease cases due to diagnostic delays contributed to higher mortality in many cohorts; however, the true impact of COVID-19 on the mortality of PC patients remains unclear and needs to be further researched [28].

In our study, age was also a major predictor of mortality. The mean age at death was 65 years, and the risk of death increased by 21% with each 10 years added to the mean age at diagnosis. Patients aged 75 and older experienced a larger increase in their mortality rate, from 31.2 in 2014 to 43.4 in 2023, compared to other age groups. These results are consistent with previous studies showing an exponential increase in the risk of death with an increasing age [30]. In the United States, between 1999 and 2020, the age-related PC mortality increased among patients aged 65 and older (63.9 per 100,000) [31]. Similarly, in China, PC patients over 60 were shown to have higher mortality rates compared to younger age groups [24]. In our data, men had a higher mortality rate compared to women (1590 vs. 1313 per 1000 PY), which is consistent with the worldwide results [32]. In a study by Didier et al., the mortality rate was higher in men than in women (12.5 versus 9.5), but both sexes showed an increase in PC deaths over recent years [31].

Furthermore, we found an overall 5-year survival rate of 10.9%, which is consistent with the world literature. Over the past 40 years, the 5-year survival rate for PC has been around 3–6%, not exceeding 10%, and no improvement in survival has been observed, unlike for other cancer types [3]. The main reason for the low survival rate is that the disease is mostly detected at inoperable stages [1,21]. Late detection due to the latent course of the disease dramatically worsens the chances of survival. When detected at stage I, the 5-year survival can reach 80% [2]. However, even after resection at early stages, PC often recurs, and the prognosis is often unfavorable [21].

As with other cancer types, screening could improve the early detection of PC and prevent high mortality. However, there is no consensus on this issue, and there are no universally accepted population-based screening programs worldwide. The research results are inconsistent, and opinions differ. Most studies suggest that cost-effective, widely used screening programs are needed to detect PC early [33]. In 2020, the American Gastroenterological Association Expert Panel recommended screening for high-risk individuals with hereditary risk factors [34]. MRI, CT, and endoscopic ultrasound (EUS) are commonly recommended as screening methods. However, all of these modalities are expensive and can be somewhat invasive for the patient, so their use in screening may be associated with complications and harm [20]. The findings suggest that further research is needed on PC screening for high-risk populations and the management of screen-detected cases.

Determining the distribution of the incidence and mortality rates across the regions of a country is important in epidemiological research [35]. According to the results of this study, the central and northern regions of Kazakhstan (Karaganda, Kostanay, North Kazakhstan, and Pavlodar) had the highest incidence rates by the end of the period. Atyrau, West Kazakhstan, Pavlodar, and Abay were characterized by the highest mortality per 1000 PY, which could have been due to the different causes. These reasons can be the different health awareness and literacy levels of the population, the level of advancement of diagnostics in the different regions, and differences in the quality of the medical care provided or access to healthcare [35]. The age structure of the population could also have had an impact on the incidence and mortality in different regions, as age is a major factor in the morbidity and mortality of PC [23].

This study has several strengths and limitations. It was the first epidemiological study in Kazakhstan on the PC incidence, mortality, and survival that utilized empirical data from the national cancer registry collected over the last 10 years. The large volume of data makes it reliable and robust. This study, like other cancer registry-based studies, has limitations. The UNEHS covers about 1/3 of the adult population of Kazakhstan, mostly urban residents and women. This could have affected the survival analysis, distorting its results. Potential confounding factors such as the cancer stage or patients’ compliance could also have affected the outcome. The lack of information on individual risk factors, such as bad habits, obesity, hereditary risk factors, etc., reduced our ability to adjust for confounding factors, and this could have led to spurious associations [16]. The strengths and limitations of the EROP as a cancer registry are discussed in a recent article by Beyembetova et al. [15]. A large cohort size is both a strength and a limitation of the EROP. It not only increases the statistical power of analyses but also increases the impact of biases and enhances the interpretation of minor findings, resulting in false conclusions. Therefore, EROP-based epidemiological studies should utilize strict methodological designs [15]. The listwise deletion of cases with missing data can reduce the statistical power and introduce bias; however, their proportion was very low in our cohort. Furthermore, the exclusion of the place of residence variable due to high missingness limited our understanding of the urban and rural cancer care in Kazakhstan.

## 5. Conclusions

PC continues to be a significant health issue worldwide, causing a heavy burden due to its high mortality. Our study revealed the global trends in Kazakhstan, as well as local regional and ethnic characteristics. The study revealed an increasing incidence of and mortality from PC in 2014–2023, very low survival rates, and inequalities based on different demographic and clinical characteristics. PC’s asymptomatic course, leading to detection at late stages, and the aggressive nature of the disease are the main reasons for its high mortality and low 5-year survival. People over 60 years of age are more susceptible to the disease, and the incidence and mortality significantly increased among them. These findings call for the implementation of measures for early diagnosis and increasing the awareness of the population. An older age, male sex, Russian ethnicity, and being from the Pavlodar and Abay regions were characterized by a higher risk of death, which may be have been due to various reasons and requires further research. This study highlights the urgent need for multilateral interventions in Kazakhstan regarding PC. Increasing the public awareness, introducing screening for high-risk individuals, and providing equal access to specialized cancer care can help improve PC’s survival and reduce its burden in Kazakhstan.

## Figures and Tables

**Table 1:** Socio-demographic and medical characteristics of patients with PC.

Characteristics	Total *n* = 11,934	Alive *n* = 1807 (15.1%)	Died *n* = 10,127 (84.9%)	*p*-Value	Mortality per 1000 PY (95% CI)
Age, mean ± SD	64.5 ± 11.3	60.2 ± 13.2	65.1 ± 10.8	<0.001	
Age group, *n* (%)				<0.001	
0–29	62 (0.5)	47 (75.8)	15 (24.2)		78 (47–130)
30–44	491 (4.2)	139 (28.3)	352 (71.7)		526 (474–584)
45–59	3032 (25.9)	475 (15.7)	2557 (84.3)		927 (892–964)
60–74	5872 (50.1)	754 (12.8)	5118 (87.2)		1334 (1298–1371)
≥75	2267 (19.3)	184 (8.1)	2083 (91.9)		1984 (1901–2071)
Sex, *n* (%)				<0.001	
Male	5669 (49.9)	522 (9.2)	5147 (90.8)		1590 (1277–1350)
Female	5636 (50.1)	673 (11.9)	4963 (88.1)		1313 (1548–1635)
Ethnicity, *n* (%)				0.011	
Other	1720 (15.2)	192 (11.2)	1528 (88.8)		1308 (1244–1375)
Kazakh	5952 (52.7)	665 (11.2)	5287 (88.8)		1414 (1377–1453)
Russian	3633 (32.1)	338 (9.3)	3295 (90.7)		1560 (1508–1615)
Clinical stage, *n* (%)				<0.001	
Stage I	429 (3.7)	252 (58.7)	177 (41.3)		159 (137–184)
Stage II	2214 (19.2)	492 (22.2)	1722 (77.8)		693 (661–726)
Stage III	4369 (37.8)	458 (10.5)	3911 (89.5)		1363 (1321–1406)
Stage IV	4224 (36.5)	206 (4.9)	4018 (95.1)		2641 (2561–2724)
Unknown	196 (1.7)	8 (4.1)	188 (95.9)		4781 (4144–5515)
Not applicable	128 (1.1)	24 (18.8)	104 (81.3)		985 (813–1193)
Tumor site in the pancreas, *n* (%)				<0.001	
Diffuse lesion (“C25”)	1007 (8.4)	118 (11.7)	889 (88.3)		864 (809–923)
Head of pancreas	7118 (59.6)	981 (13.8)	6137 (86.2)		1109 (1081–1137)
Body of pancreas	1600 (13.4)	247 (15.4)	1353 (84.6)		1140 (1081–1202)
Tail of pancreas	1070 (9.0)	188 (17.6)	882 (82.4)		1005 (941–1074)
Pancreatic duct	30 (0.3)	6 (20)	24 (80)		535 (359–799)
Endocrine part	3 (0.03)	0 (0)	3 (100)		2706 (873–8389)
Other parts	66 (0.5)	12 (18.2)	54 (81.8)		1293 (990–1688)
Overlapping sites	631 (5.3)	61 (9.7)	570 (90.3)		1651 (1521–1792)
Unspecified part	244 (2.1)	34 (13.9)	210 (86.1)		1095 (957–1254)
Benign neoplasms	165 (1.4)	160 (97)	5 (3)		14 (6–33)

**Table 2:** The relationship between the patient characteristics and PC mortality rates (2014–2023).

Variables	Crude HR (95% CI)	*p*-Value	Adjusted HR (95% CI)	*p*-Value
Age	1.21 [1.19–1.23]	<0.001	1.21 [1.19–1.24]	<0.001
Sex				
Female	1.00 (reference)		1.00 (reference)	
Male	1.13 [1.09–1.19]	<0.001	1.17 [1.06–1.20]	<0.001
Ethnicity				
Other	1.00 (reference)		1.00 (reference)	
Kazakh	0.99 [0.94–1.05]	0.725	1.05 [0.99–1.11]	0.130
Russian	1.16 [1.10–1.24]	<0.001	1.13 [1.06–1.20]	<0.001
Clinical stage				
Stage I	1.00 (reference)		1.00 (reference)	
Stage II	2.85 [2.44–3.33]	<0.001	2.15 [1.83–2.51]	<0.001
Stage III	4.58 [3.93–5.31]	<0.001	3.29 [2.82–3.83]	<0.001
Stage IV	6.66 [5.72–7.75]	<0.001	4.80 [4.13–5.59]	<0.001

## Data Availability

Due to its non-public nature, access to the dataset is limited and obtaining access requires contacting the Republican Center for Electronic Health.

## Supplementary Materials

The following supporting information can be downloaded at https://www.mdpi.com/article/10.3390/cancers17142277/s1, Figure S1: Incidence rate of pancreatic cancer in the regions of Kazakhstan by years (2014–2023). Figure S2: Kaplan-Meier survival curves of pancreatic cancer patients: (A) by age groups; (B) by sex; (C) by ethnicity; (D) by stage at diagnosis.
