# Supplementary material for: The Incidence, Mortality, and Survival Trends of Pancreatic Cancer in Kazakhstan: Data from the National Electronic Registry of Oncological Patients (2014–2023)
Source: Cancers (Basel). 2025 Jul 8;17(14):2277. doi: 10.3390/cancers17142277 (PMC12293495; doi:10.3390/cancers17142277)
Supplement: Supplementary file 1 [file cancers-17-02277-s001.zip › cancers-3660978-supplementary.pdf]

Supplementary materials

Region\year	2014	2015	2016	2017	2018	2019	2020	2021	2022	2023
Atyrau	5,3	4,5	5,1	5,2	3,8	5,6	7,3	6,1	5,9	5,1
Aktobe	3,3	4,3	3,5	7,3	4,8	6	6,6	6,6	5,5	6,1
West KZ	8,1	7	7,1	8,2	6,7	6,6	7,4	7,3	4,7	8,2
Mangystau	5	4,2	4,7	4,9	5	4,3	4,6	3,4	6,1	3,7
Karagandy	8,4	8	9,7	8,8	6,6	8	6,7	9,7	12,9	10,2
Kostanay	6,6	7,7	8,8	10,2	11,7	9,1	9,4	8,6	9,3	10,9
Akmola	10,7	7,2	11,9	11,1	8,8	12,9	8,3	9,6	9,5	8,6
city Astana	4,8	5,8	7,8	5,5	7,3	6,7	7,5	6,6	6,8	7,2
North KZ	8,7	9,5	10,6	11,1	9,7	12,7	11,7	7	11	10,2
Pavlodar	10	8	6,8	8,8	12	11,9	8,7	9,9	11,7	10,5
East KZ	9,3	8,4	7,9	9,9	9	9,2	7,9	9,9	9,6	9,9
Almaty	4,1	5,1	4,1	5,6	4,6	4,4	4,7	4,7	4,3	4,2
city Almaty	6,8	6,1	7,4	6,9	10,1	7,3	7,8	8,2	7,4	7,5
Zhambyl	3,8	3,1	4,4	4,9	4,7	4,3	6,7	6,3	6	4,2
Kyzylorda	3,3	4	5,7	6,1	5,2	5	5,7	5,2	5,6	4
South KZ	2,9	3,7	3,9	3,8						
city Shymkent					9	5,1	5,6	4,1	3,7	5
Turkistan					0,7	2,7	2,3	2,6	2,7	3,2
Zhetissu									4,8	8,8
Ulytau									1,4	5,5
Abay									6,4	10

Note. The color scale ranges from blue (low incidence) to red (high incidence); KZ, Kazakhstan.

Figure S1. Incidence rate of pancreatic cancer in the regions of Kazakhstan by years (2014–2023).

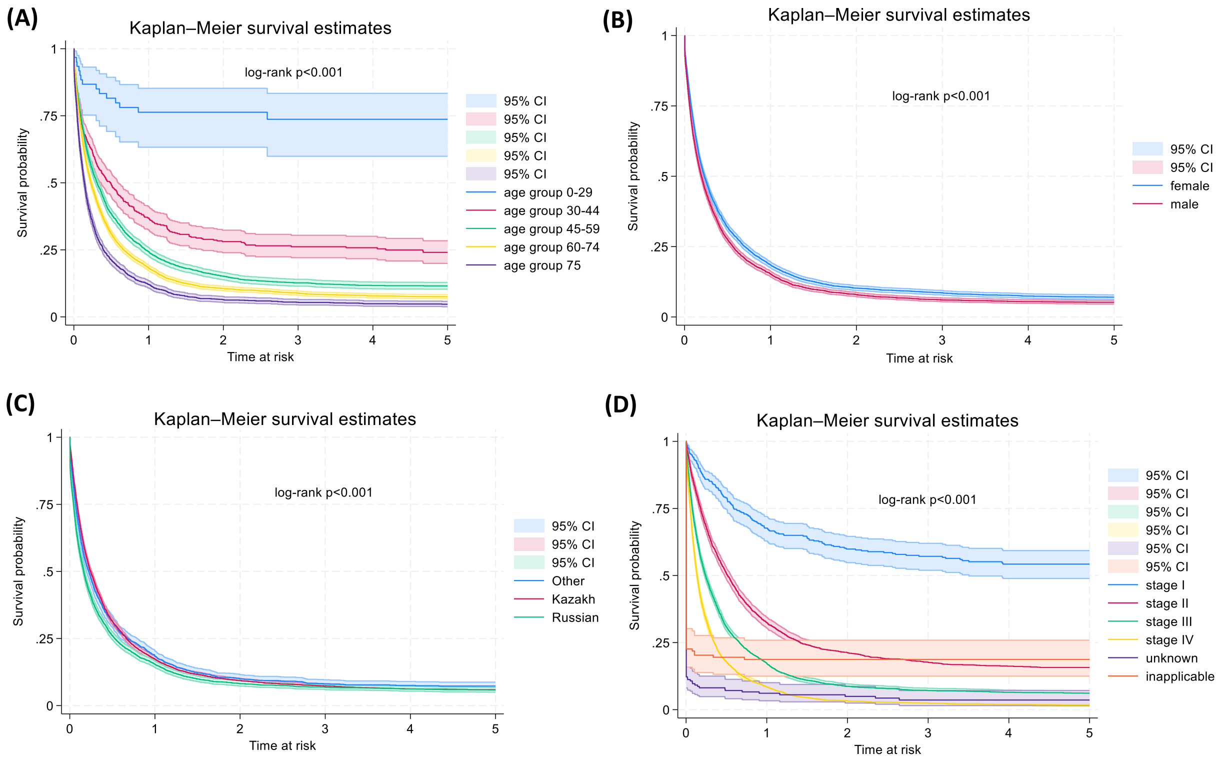

Figure S2. Kaplan-Meier survival curves of pancreatic cancer patients: (**A**) by age groups; (**B**) by sex; (**C**) by ethnicity; (**D**) by stage at diagnosis.
